# A Histological Evaluation of Artificial Dermal Scaffold Used in Micrograft Treatment: A Case Study of Micrograft and NPWT Performed on a Postoperative Ulcer Formation after Tumor Resection

**DOI:** 10.3390/medicina58010073

**Published:** 2022-01-04

**Authors:** Yuta Niimi, Kyoko Baba, Masako Tsuchida, Akira Takeda

**Affiliations:** 1 Department of Plastic Surgery, Kitasato University Medical Center, Saitama 364-8501, Japan; y.niimi@kitasato-u.ac.jp (Y.N.); masako19@kitasato-u.ac.jp (M.T.); takeda@kitasato-u.ac.jp (A.T.); 2 Department of Plastic and Aesthetic Surgery, School of Medicine, Kitasato University, Kanagawa 252-0373, Japan

**Keywords:** micrograft, artificial dermis, negative pressure wound therapy, skin ulcer, histological evaluation

## Abstract

*Background and Objectives*: Wound healing (WH) is a complex natural process: the achieving of a proper WH with standard therapies sometimes is not fulfilled and it is often observed in aged and diabetic patients, leading to intractable ulcers. In recent years, autologous micrograft (AMG) therapies have become a new, effective, and affordable wound care strategy among both researchers and clinicians. In this study, a 72-year-old female patient underwent a combination of treatments using micrograft and negative pressure wound therapy (NPWT) on a postoperative skin ulcer after a benign tumor resection on the back with the aim to present an innovative method to treat skin ulceration using AMG combined with an artificial dermal scaffold and NPWT. *Materials and Methods*: A section of the artificial dermal scaffold, infused with micrografts, was sampled prior to transplant, and sections were collected postoperatively on days 3 and 7. Hematoxylin-eosin (HE) and immunohistochemical stains were employed for the evaluation of Cytokeratin AE1/AE3, desmin, and Factor VIII. Additionally, on postoperative day 3, NPWT dressing was evaluated using HE stains, as well. The resulting HE and immunostaining analysis revealed red blood cells and tissue fragments within the collagen layers of the artificial dermis prior to transplant. On postoperative day 3, collagen layers of the artificial dermis revealed red blood cells and neutrophils based on HE stains, and scattering of cytokeratin AE1/AE3-positive cells were detected by immunostaining. The HE stains on postoperative day 7 showed more red blood cells and neutrophils within the collagen layers of the artificial dermis than on day 3, an increase in cytokeratin AE1/AE3-positive cells, and tissue stained positively with desmin and Factor VIII. *Results*: Results suggest that the effects of both micrografts and migratory cells have likely accelerated the wound healing process. Furthermore, the NPWT dressing on day 3 showed almost no cells within the dressing. This indicated that restarting NPWT therapy immediately after micrograft transplant did not draw out cells within the scaffold. *Conclusions*: Micrograft treatment and NPWT may serve to be a useful combination therapy for complex processes of wound healing.

## 1. Introduction

Wound healing (WH) is a complex natural process requiring different cellular components and molecular pathways. The achieving of a proper WH sometimes is not fulfilled and it is often observed in aged and diabetic patients, leading to intractable ulcers. Intractable ulcers have a significant impact in terms of quality of life and require significant efforts and complex management.

Standard therapies, such as continuous debridement, compression, wound dressing, local or systemic antibiotics, and venous surgery, have been extensively investigated. In recent years, autologous micrograft (AMG) therapies have become a new, effective, and affordable wound care strategy among both researchers and clinicians [1].

Furthermore, micrografting promptly answers to the main limitations distinguishing the gold standard method of autologous grafting (AG), namely the necessity of a large amount of tissue, and the consequence of long-term hospitalization [2].

In this study report, we describe the use of a micrograft technology to treat a 72-year-old female patient, affected by an intractable ulcer after tumoral resection. Specifically, Rigenera^®^ technology allows the mechanical disaggregation of a small quantity of tissue, directly harvested from the patient, from which micrografts of about 80 µm are obtained through a medical device called Rigeneracons. Micrografts are enriched in progenitor cells expressing MSC-like markers, providing strong regenerative potential [2].

In addition to this micrograft technology, an artificial dermis (Pelnac G plus^®^) was used as a scaffold wherein micrografts have been placed to cover the wound [3,4,5,6]. This called into question whether the transplanted tissue fragments remained within the scaffold with certainty, and what changes occurred over time within the scaffold. With the intention of shedding light on these questions, we have histologically evaluated the artificial dermal scaffold used for the micrograft treatment.

Finally, to support the wound healing process, negative pressure wound therapy (NPWT), and patch graft were performed to provide a good environment and epidermal tissue.

Hence, this work aims to present, characterize, and evaluate a new and innovative method to treat skin ulceration using autologous micrograft combined with an artificial dermal scaffold, NPWT, and patch graft.

## 2. Materials and Methods

**Figure 1:**
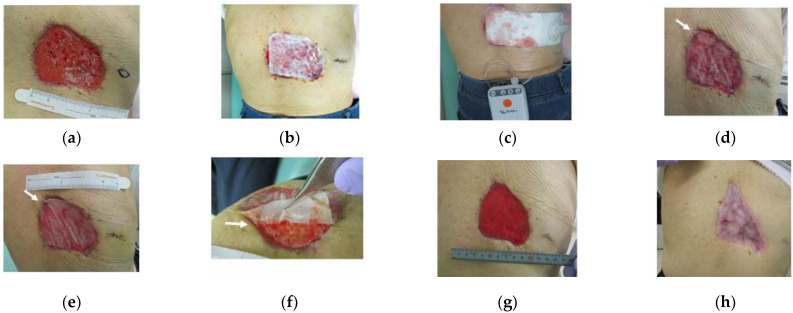
Case: 72-year-old female patient with a post-surgical skin ulcer after a tumor resection on the back. (**a**) Preoperative observation: ulcer formation was 80 mm × 85 mm in size. Donor sample was harvested from the back near the skin defect, excised in a spindle shape with the longer diameter measuring 15 mm. (**b**) Immediately after application of artificial dermis: micrograft-infused artificial dermis was applied to the wound. (**c**) NPWT: Immediately after the operation, NPWT was started using PICO 7^®^. (**d**) Postoperative day 3: At the time of transplant, the section (shown with arrow) of the artificial dermis was adjacent to the wound but became unnecessary due to ulcer contraction and was collected for evaluation. (**e**) Postoperative day 7: The artificial dermis was still close to the wound on postoperative day 3, but this section (shown with arrow) became unnecessary due to ulcer contraction and was collected for evaluation. (**f**) Postoperative day 7: The silicone film of the artificial dermis was removed. Almost all of the collagen sponge layer adhered to the ulcer surface, and the silicone film was easily removed. (**g**) Overall contraction of the ulcer was achieved with favorable granulation. (**h**) Epithelialization of the ulcer.

The case subject is a 72-year-old female patient. The patient’s medical history consists of heart failure, type 2 diabetes, chronic renal failure (hemodialysis patient), and hypothyroidism. After resection of eccrine poroma located on the back, the patient formed a skin ulcer, 80 mm × 85 mm in size (Figure 1a). The ulceration was treated with autologous micrograft (Rigenera^®^, HBW srl, Turin, Italy), artificial dermis (Pelnac G Plus^®^, Gunze Limited, Kyoto, Japan) and NPWT (PICO™ 7, Smith & Nephew plc, London, UK).

Samples of the artificial dermis, used as a scaffold for the micrograft, and NPWT dressing were collected to confirm the presence of the micrografts within the scaffold and to evaluate its changes over time.

### 2.1. Preparation of Micrograft

Rigenera^®^ technology allows to obtain AMG through a disposable medical device called Rigeneracons, whereby the patient is both the donor and the recipient. For this case, the tissue donor sample was harvested from the back near the skin defect and excised with a surgical blade in a spindle shape specimen with the longer diameter measuring 15 mm (Figure 1a).

**Figure 2:**
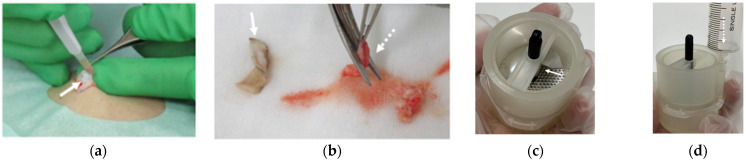
Preparation of the micrograft (using Rigenera^®^ system and Rigeneracons). (**a**) Harvesting of tissue: The tissue sample was harvested and divided into two layers, the epidermal layer and the dermal-subdermal layer. The arrow shows the epidermis collection from the superficial layer of the dermis. (**b**) Harvested tissue: Epidermal layer (solid arrow) and the dermal-subdermal layer (dashed arrow) are shown. The tissue was cut into 2–3 mm size using a scissor. (**c**) Sample tissues are placed inside the Rigeneracons (solid arrow). (**d**) 16 mL physiological saline solution is added from the side hole (dashed arrow). Micrograft suspension is prepared by cutting tissues into fragments of 80 μm using the Rigenera^®^ system (photo not shown).

The tissue sample was then divided into two layers, the epidermal layer and the dermal-subdermal layer (Figure 2a,b). Using Rigeneracons (Rigenera^®^, HBW srl, Turin, Italy), a micrograft suspension was acquired through a mechanical action and without the addition of enzymes or any chemical substances. The device is a sterile capsule characterized by a grid with hexagonal holes equipped with steel blades rotating at 80 rpm and activated by an engine. Preparation required cutting the tissue sample into smaller pieces of 2–3 mm in size (Figure 2b) which are then placed inside the Rigeneracons (Figure 2c) along with 16 mL physiological saline solution (Figure 2d). The entire tissue disaggregation took only 2 min and 30 s.

### 2.2. Micrograft Infusion into Artificial Dermis and Transplantation

After cleansing the wound surface with physiological saline solution, the dermal-subdermal micrograft suspension was transplanted by injecting it into the ulcer and under the skin of the ulcer margin.

**Figure 3:**
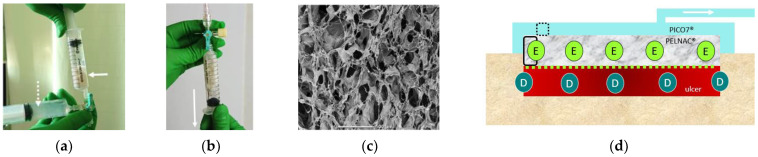
Infusion of micrografts into artificial dermis & schematic diagram of the wound site. (**a**) Micrograft suspension derived from the epidermal layer (dashed arrow) was placed inside a syringe with artificial dermis (PELNAC G plus^®^) (solid arrow) for infusion. The epidermal micrograft suspension (dashed arrow) was turbid and slightly viscous. (**b**) The syringe plunger is pulled in the arrow direction to apply negative pressure of approximately 0.5 atm in order to infuse the artificial dermis (PELNAC G plus^®^) with epidermal micrograft suspension. (**c**) SEM (Scanning Electron Microscope) image of the collagen sponge layer in the artificial dermis (PELNAC G plus^®^). (**d**) Schematic diagram of the ulcer immediately after the treatment: the dermal-subdermal micrograft (D) suspension is sub-dermally injected in the ulcer and around the ulcer margin for transplant. The artificial dermis infused with epidermal micrograft (E) was applied to the wound. Immediately thereafter, NPWT was performed. Solid line area shows the sample section of the artificial dermis, and the dashed line area represents the sample section of the dressing.

Subsequently, the epidermal micrograft suspension (Figure 3a) was placed inside a syringe with a two-layered artificial dermis which is comprised of collagen sponge and silicone film (PELNAC G plus^®^, Gunze Limited, Kyoto, Japan). A negative pressure of 0.5 atm was then applied (Figure 3b) to infuse the collagen sponge (Figure 3c) with micrografts.

After spraying the remaining suspension onto the ulcer surface, the micrograft-infused artificial dermis was applied to the wound (Figure 1b). Immediately after the treatment, NPWT was started using PICO 7^®^ wound therapy system (Smith & Nephew plc, London, UK) (Figure 1c).

A schematic diagram is provided in Figure 3d for reference.

### 2.3. Histological Examination

Sections of the micrograft-infused artificial dermis, that which are commonly discarded, were collected prior to the transplant, postoperatively on day 3 (Figure 1d) and on day 7 (Figure 1e).

HE staining and immunostaining (cytokeratin AE1/AE3 for epithelial cells; desmin for smooth muscles and myofibroblasts; Factor VIII for vascular endothelial cells) were performed for histological evaluations.

Although the samples collected on the 3rd and the 7th day post-surgery were adjacent to the ulceration, the areas where sampling was performed were assessed as being unnecessary for the healing process (Figure 1d,e) due to the contraction of the wound.

In addition, a section of the NPWT dressing was obtained on day 3 during its first dressing change for HE stains assessment.

### 2.4. Follow-Up after the Micrograft Transplant

The silicone film was removed from the artificial dermis postoperatively on day 7 (Figure 1f), showing favourable granulation (Figure 1g).

Despite the formation of granulation tissue, the ulcer area was large with considerable epithelial defect and epithelialization was expected to take long. Therefore, a patch graft was performed and NPWT was concluded on the 27th day from the initial micrograft treatment.

Complete epithelialization was observed postoperatively on day 54 (Figure 1h), and no complications were reported during the follow-up period.

## 3. Results

### 3.1. HE Staining of the Artificial Dermal Scaffold

**Figure 4:**
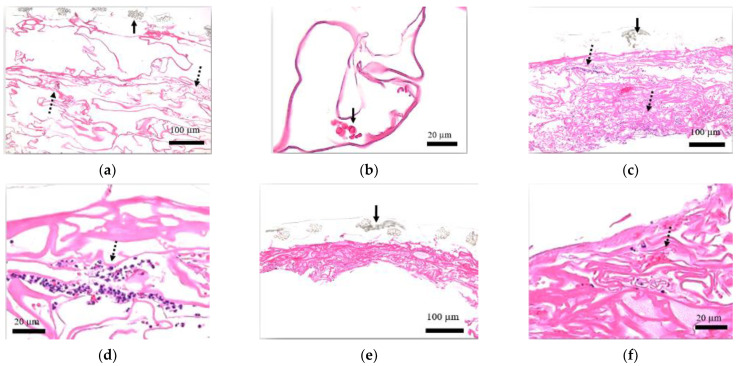
HE evaluation of artificial dermis sample. (**a**) Low power field of view before transplant (40×): Confirmed tissue fragments (dashed arrows) inside the collagen sponge. Solid arrow represents silicone film. (**b**) High power field of view before transplant (200×): Red blood cells (solid arrow) found within the artificial dermis were regarded as influence from the sampling procedure. (**c**) Low power field of view postoperative day 3 (40×): Confirmed a predominance of neutrophils inside the collagen sponge (dashed arrows). Solid arrow represents silicone film. (**d**) High power field of view postoperative day 3 (200×): Confirmed a predominance of neutrophils inside the collagen sponge (dashed arrow). (**e**) Low power field of view postoperative day 7 (40×): Various cells and blood cells, including neutrophils, were detected. In addition, the collagen sponge layer changed over time and became compressed. Solid arrow represents silicone film. (**f**) High power field of view postoperative day 7 (200×): Within the collagen sponge, some blood cells were surrounded by cells with flat nucleus, indicating capillary-like tissues (dashed arrow).

From the sampling prior to transplant, the presence of micrograft was confirmed inside the artificial dermis (Figure 4a,b). Aside from that, blood cells found within the artificial dermis were regarded as influence from the sampling procedure.

Day 3 from the treatment showed a predominance of neutrophils inside (Figure 4c,d). Various cells and blood cells, including neutrophils, were detected on day 7; among the blood cells, some were observed to have a flat nucleus, indicating capillary-like tissues (Figure 4e,f).

In addition, histological evaluation showed that the collagen sponge layer changed over time and became compressed.

### 3.2. Staining of Cytokeratin AE1/AE3

**Figure 5:**
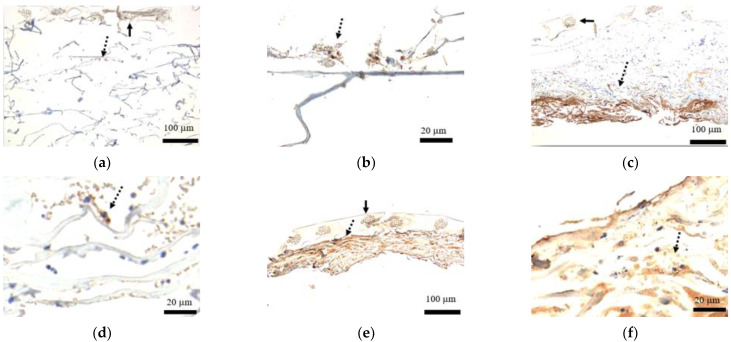
Staining of cytokeratin AE1/AE3 within the artificial dermal scaffold. (**a**) Low power field of view before transplant (40×): Cytokeratin AE1/AE3 expression was found on a tissue within the artificial dermis (dashed arrow). Solid arrow represents silicone film. (**b**) High power field of view before transplant (200×): Cytokeratin AE1/AE3 expression was found on a micrograft within the artificial dermis (dashed arrow). (**c**) Low power field of view postoperative day 3 (40×): Cells stained with cytokeratin AE1/AE3 were predominant on the ulceration side (dashed arrow). Solid arrow represents silicone film. (**d**) High power field of view postoperative day 3 (200×): Cells stained with cytokeratin AE1/AE3 expression were found in the collagen sponge (dashed arrow). (**e**) Low power field of view postoperative day 7 (40×): The presence of cells with cytokeratin AE1/AE3 expression has increased (dashed arrow) and was detected more on the silicone film side. Solid arrow represents silicone film. (**f**) High power field of view postoperative day 7 (200×): A number of cells stained with cytokeratin AE1/AE3 expression were found in the collagen sponge (dashed arrow).

Cytokeratin AE1/AE3 expression was found on a tissue inside the artificial dermis prior to transplant (Figure 5a,b). Postoperatively on day 3, cells stained with cytokeratin AE1/AE3 were predominant on the ulceration side (Figure 5c,d), and on day 7, the presence has increased and was seen also on the silicone film side (Figure 5e,f).

### 3.3. Staining of Desmin

**Figure 6:**
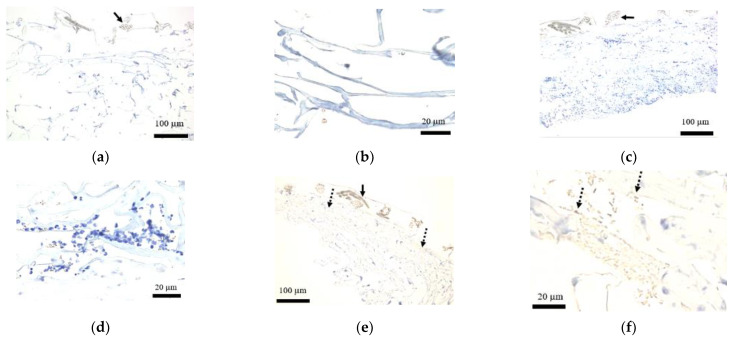
Staining of desmin within the artificial dermal scaffold. (**a**) Low power field of view before transplant (40×): No tissue within the collagen sponge was stained with desmin. Solid arrow represents silicone film. (**b**) High power field of view before transplant (200×): No tissue within the collagen sponge was stained with desmin. (**c**) Low power field of view postoperative day 3 (40×): No tissue within the collagen sponge was stained with desmin. Solid arrow represents silicone film. (**d**) High power field of view postoperative day 3 (200×): No tissue within the collagen sponge was stained with desmin. (**e**) Low power field of view postoperative Day 7 (40×): A slight positivity with desmin was seen within part of the collagen sponge (dashed arrow). Uneven distribution was not seen among the stained cells. Solid arrow represents silicone film. (**f**) High power field of view postoperative day 7 (200×): A slight positivity with desmin was seen within part of the collagen sponge (dashed arrows).

Prior to transplant and postoperatively on day 3, the analysis of artificial dermis indicated no tissue staining with desmin (Figure 6a,d). On day 7, part of the artificial dermis showed a slight positivity with desmin (Figure 6e,f). Uneven distribution was not seen among the stained cells.

### 3.4. Staining of FactorVIII

**Figure 7:**
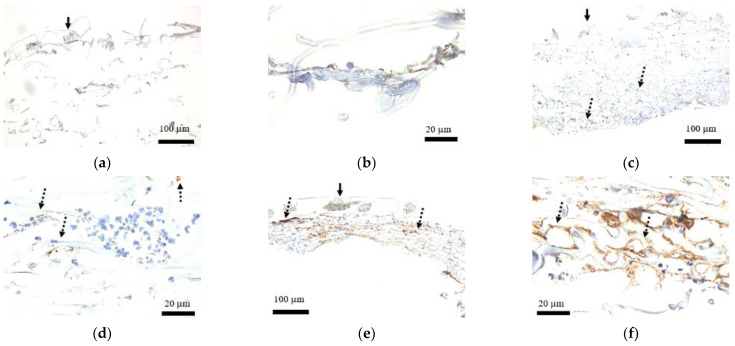
Staining of Factor VIII within the artificial dermal scaffold. (**a**) Low power field of view before transplant (40×): Blood cells were detected within the collagen sponge; however, no tissue was positive with Factor VIII. Solid arrow represents silicone film. (**b**) High power field of view before transplant (200×): Blood cells were detected within the collagen sponge; however, no tissue was positive with Factor VIII. (**c**) Low power field of view postoperative day 3 (40×): Some cells were stained with Factor VIII (dashed arrows). Solid arrow represents silicone film. (**d**) High power field of view postoperative day 3 (200×): Some cells were stained with Factor VIII (dashed arrows). (**e**) Low power field of view postoperative day 7 (40×): An increase in Factor VIII-stained cells were seen (dashed arrows). Solid arrow represents silicone film. (**f**) High power field of view postoperative day 7 (200×): Part of the cells stained with Factor VIII formed lumen-like structures (dashed arrows).

Although blood cells were indicated within the artificial dermis before transplant, no tissue was positive with Factor VIII (Figure 7a,b). Postoperatively on day 3, some cells were stained with Factor VIII (Figure 7c,d), and an increase of presence was seen on day 7, with some cells forming a lumen-like structure (Figure 7e,f).

### 3.5. NPWT Dressing on Postoperative Day 3

**Figure 8:**
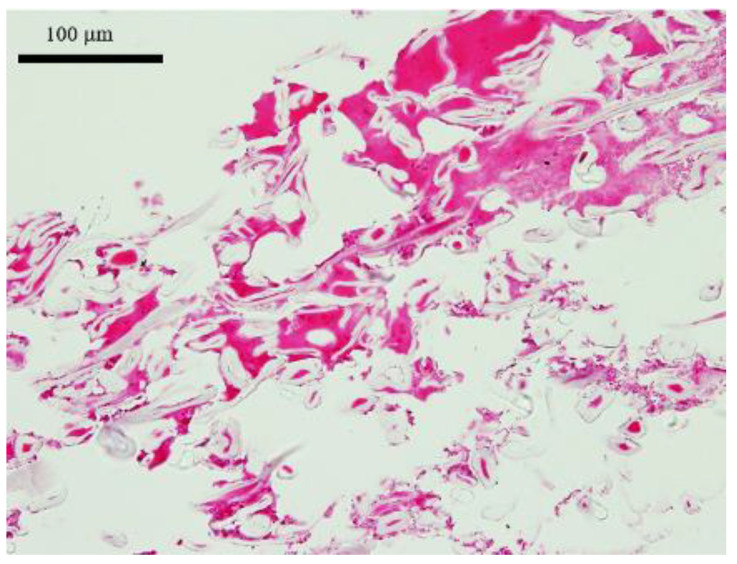
HE assessment of NPWT dressing. On postoperative day 3, a part of the NPWT dressing was obtained for HE stain assessment during its first dressing change. The dressing contained inflammatory cells and blood cells, but no cells were seen as being leaked from the skin grafts.

In order to find out whether the micrograft leaked from the artificial dermal scaffold, NPWT dressing was examined. Upon observation from the HE staining, the inside of the dressing contained blood cells and inflammatory cells, but no cells were seen as being derived from micrografts (Figure 8).

## 4. Discussion

### 4.1. The Micrograft Concept

The micrograft technique is an application that was first introduced by Reverdin in 1869 as a transplantation method using full thickness skin that is cut into small grafts for wound healing [7]. Since then, variations of this technique in different graft sizes came about, called under different names, such as, pinch graft, patch, postage stamp graft, and scrap graft [7,8,9,10,11,12]. In the 1980′s, micrograft started to gain attention again, and the micrograft technique reported by Zhang et al. [13,14] became the basis of what is currently utilized.

In 2013, HBW srl (Italy) created the Rigenera^®^ technology, which allows to obtain fragments of about 80 µm from autologous tissue sample using a medical device called Rigeneracons. In 2015, Trovato et al. [2] demonstrated its efficacy through a preliminary in vitro study. Findings by Jimi et al. in 2017 describes an in vivo study using mice for the histological assessment of micrografts [15].

Another study discussing the micrograft concept using autologous tissue for wound healing, published in 2010, by Biswas et al. [12] has been referenced and summarized under Table 1, with new information added to the prior micrograft studies.

Based on reports from Trovato et al. [2] and Jimi et al. [15], the Rigenera^®^ system, which we have used in our case, allows the mechanical disaggregation of several types of tissues achieving fragments with an approximate size of 80 µm, suspended in physiological solution. This autologous tissue suspension can be transplanted to attain acceleration of wound healing.

According to the study by Jimi et al. [15], TGF-β1 (transforming growth factor-β1) expression was upregulated in granulation in its early phase after the transplant. This was followed by an increase in the number of myofibroblasts expressing αSMA (α-smooth muscle actin), with neovascularization and collagen scaffold maturation, inducing the granulation tissue to thicken and contract, and accelerating the wound healing process. In the same study, GFP (Green Fluorescent Protein) was used in the experiment to ascertain whether the micronized tissue fragments multiply after transplantation; findings indicated that after playing an initial role in the granulation tissue formation, the grafted epidermal cells undergo apoptosis.

In another experiment conducted on mice, Jimi et al. [18] describes that the micrografts help acceleration of the wound through angiogenesis. It can be surmised that a similar mechanism may take place in humans, but due to ethical considerations, histological assessments based on clinical evidence to determine the promotion of wound healing mechanism are rare. Having said that, many clinical cases reported favourable outcomes [4,19], leading the Rigenera^®^ technology to be introduced and used in several fields, including plastic and reconstructive surgery, orthopaedic surgery, and aesthetic cosmetic surgery.

### 4.2. In Consideration of Our Case Study

We were able to confirm the presence of micrograft within the artificial dermal scaffold. The structure of PELNAC G plus^®^ scaffold is a two-layered artificial dermis. The first layer consists of a collagen sponge of alkali-treated gelatin which provides a scaffold for the tissue regeneration, and the second layer is a fortified silicone film. Through the application of negative pressure, the cavities within the collagen sponge layer demonstrated that micrograft suspension was infused inside the scaffold. Cavities within the artificial dermis are known to provide a foothold for capillary vessels and fibroblast cells in the tissue regeneration process, and we have considered this to be an effective technique for our case.

Results from the immunostaining evaluation showed that on postoperative day 7, the artificial dermis had neovascularization, which was not evident postoperatively on day 3. In other words, the scaffold contained non-epithelial-derived cells, such as neutrophils, collagen fiber, and vascular endothelial cells, all of which gradually increased over time. Particularly in relation to the increase of neutrophils, migration from the graft bed to the artificial dermis was thought to be more likely the cause. Due to the lack of presence of phagocytes, the migration was considered to be not caused by a foreign body reaction, but rather associated with an inflammatory reaction in the wound healing process, in which the micrografts may have triggered a mediator production.

Typically, during a wound healing process several factors are involved, among them the TGF-β1 is activated early on, followed by collagen production and angiogenesis. Through the histological examination of the artificial dermis, the above-mentioned expected parameters were reasonably confirmed, and appeared consistent with the findings of Jimi et al. [15], as well.

In our clinical case, we cannot specify whether the cellular inflammatory response within the artificial dermis was induced by the presence of micrografts, or whether it was due to cell’s migration from the ulcer surface. Considering the process of wound healing, both epidermal and dermal tissue fragments were infused into the scaffold, leading to estimate a migration of neutrophils. Alternately, epithelial cells and fibroblasts could be micrograft-derived.

From this understanding, we speculate that the micrograft and migratory cells are involved in progression and facilitation of the wound healing process. This may signify that micrografts themselves do not multiply but release some factors capable of influencing the surrounding environment by facilitating the cells migration and inducing angiogenesis.

Based on mice experiments performed by Jimi et al. [15], we may reasonably hypothesize that our clinical case resembles their outcomes, suggesting that our histological assessment inside the scaffold points to similar reactions within the adjacent ulcer. Despite the difficulty to obtain a patient’s tissue to perform histological assessment, except for exclusively therapeutic purposes, our clinical case is in line with this perspective, and are consistent with findings from previous studies.

When the micrograft stimulates wound healing, the transplanted tissue survives in order to play a role based on its function. Trovato et al. [2] demonstrated that the Rigenera^®^ system is able to create tissue suspension within a short period of time, allowing for longer cell viability within the tissue suspension. Although we cannot clinically trace the micrograft after transplant, we have been able to prepare micrograft within a short time period, and the results were favourable as seen from the histological assessments.

Furthermore, no cell leakage was evident from the NPWT dressing after transplant. This indicates that the usage of NPWT immediately after the transplant does not compromise the function of micrografts that reside in the artificial dermis.

## 5. Conclusions

We have conducted micrografts treatment and NPWT on an ulcer formed after tumor resection, with favourable results. For this case, a histological assessment was performed on the artificial dermis which was used as a scaffold for the micrografts, and it implies that micrografts and migratory cells are involved in progression and facilitation of the wound healing process. Finally, when employing an artificial dermis as a scaffold, the usage of NPWT immediately after transplant did not seem to affect the outcome.

## Figures and Tables

**Table 1:** Transition from skin grafting to micrograft [12].

Published Year & Author	Name of Graft Technique	Graft Size	Expansion Ratio	Cutting Method
1869: JL Reverdin	Pinch graft	2–5 mm^2^	6–7	Lifting the epidermis with a needle point and cutting the lifted epidermis with a scalper for harvesting.
1943: P Gabbaro	Patch graft/ Scrap graft	1.27 mm^2^ Various sizes	6–9	Donor skin that is one-sixth to one-ninth the size of the wound is placed on paper with dermis side up, and cut into small squares. A modified method uses a split-thickness skin graft, cutting them into small postage stamp sizes, known as “scrap grafts”.
1958: CP Meek	Meek microdermagraft	1.58 mm^2^	9	Placing the graft on a cork carrier to create microskin grafts using the Meek-wall Microdermatome.
1986: ML Zhang et al.	Microskin graft	1 mm^2^	7–100	Skin is minced with scissors into pieces smaller than 1 mm^2^.
1987: SD Blair et al.	Microscopic split-skin graft	40–200 µm^2^	20–26	The technique uses a histological tissue slicer to prepare diced grafts that are 200 µm^2^ in area.
1993: RW Kreis et al.	Modified Meek Technique	9 mm^2^	10	Modified meek-wall dermatome.
2000: SS Lee et al.	Modified postage stamp graft	25 mm^2^	9	The skin is placed on stainless-steel plates called “quick cutting plates” for cutting the graft.
2002: W Xie et al.	Microskin grafting by spraying	0.04–0.25 mm^2^	110–150	Harvested tissue fragment is cut into pieces of 0.2–0.5 mm in size.
2009: Y Rissin et al.	Modified skin suspension technique	0.4 mm^2^	15	Pulse blending the skin and then spreading the particles over a synthetic fenestrated Telfa sheets.
2015: L Trovato et al.	Micrograft technique	80 µm	20	Disaggregation into 80 µm size using Rigenera^®^ system [16,17]

## Data Availability

All the data are available from the corresponding author upon reasonable request.
